# Maternal use of methamphetamine induces sex‐dependent changes in myocardial gene expression in adult offspring

**DOI:** 10.14814/phy2.15509

**Published:** 2022-11-25

**Authors:** Alex Dague, Hasitha Chavva, Daniel A. Brazeau, James Denvir, Boyd R. Rorabaugh

**Affiliations:** ^1^ Department of Pharmaceutical Sciences Marshall University School of Pharmacy Huntington West Virginia USA; ^2^ Department of Biomedical Science Marshall University School of Medicine Huntington West Virginia USA

**Keywords:** drug abuse, fetal reprogramming, heart, pregnancy, prenatal methamphetamine

## Abstract

Methamphetamine is a commonly abused illicit stimulant that has prevalent use among women of child‐bearing age. While there are extensive studies on the neurological effects of prenatal methamphetamine exposure, relatively little is known about the effect of prenatal methamphetamine on the adult cardiovascular system. Earlier work demonstrated that prenatal methamphetamine exposure sex dependently (females only) sensitizes the adult heart to ischemic injury. These data suggest that prenatal exposure to methamphetamine may induce sex‐dependent changes in cardiac gene expression that persist in adult offspring. The goal of this study was to test the hypothesis that prenatal methamphetamine exposure induces changes in cardiac gene expression that persist in the adult heart. Hearts of prenatally exposed female offspring exhibited a greater number of changes in gene expression compared to male offspring (184 changes compared with 74 in male offspring and 89 changes common between both sexes). Dimethylarginine dimethylaminohydrolase 2 and 3‐hydroxybutyrate dehydrogenase 1 (genes implicated in heart failure) were shown by Western Blot to be under expressed in adult females that were prenatally exposed to methamphetamine, while males were deficient in 3‐Hydroxybutyrate Dehydrogenase 1 only. These data indicate that prenatal methamphetamine exposure induces changes in gene expression that persist into adulthood. This is consistent with previous findings that prenatal methamphetamine sex dependently sensitizes the adult heart to ischemic injury and may increase the risk of developing cardiac disorders during adulthood.

## Graphical abstract

Methamphetamine is a commonly abused illicit stimulant that has prevalent use among women of child‐bearing age. While there are extensive studies on the neurological effects of prenatal methamphetamine exposure, relatively little is known about the effect of prenatal methamphetamine on the adult cardiovascular system. These data indicate that prenatal methamphetamine exposure induces changes in gene expression that persist into adulthood.

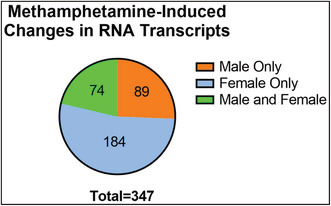

## 1 INTRODUCTION

Methamphetamine is one of the most commonly used illicit drugs in the United States. The 2020 National Survey on Drug Use and Health estimated that more than 2.5 million US residents over the age of 12 have used methamphetamine during the past year (Substance Abuse and Mental Health Services Administration, 2021). This represents a 48% increase in methamphetamine use compared with the same annual survey conducted 5 years earlier (Center for Behavioral Health Statistics and Quality, 2018). Methamphetamine use is most prevalent among adults 26–49 years of age (Substance Abuse and Mental Health Services Administration, 2021). Importantly, this includes women of childbearing age. The Infant Development, Environment, and Lifestyle (IDEAL) study estimated that 5.2% of pregnant women used methamphetamine during their pregnancy (Arria et al., 2006) and that most women who regularly used methamphetamine prior to pregnancy continued to use this drug after recognizing that they were pregnant (Della Grotta et al., 2010; Wright et al., 2015).

Most studies investigating the impact of methamphetamine use during pregnancy have focused on neurological, cognitive, and behavioral outcomes in the offspring (Chang et al., 2004; Diaz et al., 2014; LaGasse et al., 2012; Roos et al., 2014). In contrast, few studies have investigated the impact of prenatal exposure to methamphetamine on adult cardiovascular function. We recently reported that prenatal exposure to methamphetamine induces sex‐dependent changes in the cardiovascular system that extend into adulthood (Chavva et al., 2022). Adult male rats (but not their female littermates) that were prenatally exposed to methamphetamine exhibit vascular dysfunction that is characterized by potentiation of angiotensin II‐induced contraction of the aorta, attenuation of acetylcholine‐induced relaxation, and alterations in the function of perivascular adipose tissue that plays an important role in regulating vascular tone (Chavva et al., 2022). We have also found that adult female rats (but not their male littermates) that were prenatally exposed to methamphetamine developed myocardial hypersensitivity to ischemic injury (Rorabaugh et al., 2017). This was accompanied by decreased expression of protein kinase C‐ε and decreased phosphorylation of Akt, proteins that have well established roles in protecting the heart from ischemic injury (Bell et al., 2007; Downey et al., 2007; Hausenloy et al., 2005). These data suggest that individuals that were prenatally exposed to methamphetamine may be at increased risk of developing cardiovascular diseases when they become adults.

The concept that exposure to an adverse uterine environment (such as fetal hypoxia or malnutrition) can lead to long‐term changes in gene expression and increased susceptibility to disease during adult life is well established (Barker et al., 1989; Bourque et al., 2013; Bourque & Davidge, 2020; de Boo & Harding, 2006). Our observation that prenatal methamphetamine induces cardiovascular changes that persist into adulthood suggests that the maternal use of methamphetamine may induce long‐term changes in gene expression in the heart and vasculature of the offspring. Thus, the goal of the present study was to determine whether prenatal exposure to methamphetamine induces changes in myocardial gene expression that persist into adulthood. This is the first study to demonstrate that methamphetamine use during pregnancy induces global changes in cardiac gene expression in adult offspring and that adult female offspring are more susceptible than adult male offspring to changes in cardiac gene expression following methamphetamine exposure during the gestational period.

## 2 METHODS

### 2.1 Animals and drug treatment

Male and female Sprague–Dawley rats were co‐housed in standard cages with free access to food and water on a 12/12 h light/dark cycle (lights on at 0600). Females were monitored twice per day (06:00 and 18:00) for vaginal plugs, and the presence of a vaginal plug was considered as gestational Day 0. Pregnant rats were administered saline (3 pregnant dams) or methamphetamine (4 pregnant dams) by subcutaneous injection (5 mg/kg/day) once per day (at 0800) starting at gestational Day 1 and continuing until the pups were born. This dose of methamphetamine is within the range of methamphetamine doses typically used by people in illicit settings (McKetin et al., 2006). In addition, this dose of methamphetamine has been shown to hypersensitize the adult heart to myocardial ischemic injury and is commonly used by other investigators to study the behavioral and locomotor effects of methamphetamine The pups were weaned at postnatal Day 28 and housed two animals per cage. All procedures were approved by the Institutional Animal Care and Use Committee of Marshall University.

### 2.2 Heart isolation and RNA isolation

Adult offspring were anesthetized with pentobarbital (150 mg/kg) at 8 weeks of age. The hearts were quickly mounted on a Langendorff isolated heart system and perfused with Krebs solution for 5 min to flush blood from the tissue. Hearts were then flash frozen in liquid nitrogen and stored at −80°C. All heart isolations occurred between 0900 and 1100 in the morning.

Total RNA was isolated using Trizol (Thermo Fisher) according to the manufacturer's instructions. RNA integrity was confirmed prior to library construction using an Agilent 2100 bioanalyzer to confirm that all RNA samples had RNA integrity values greater than 8. Offspring from each of the saline (3 pregnant saline‐treated dams) or methamphetamine (4 pregnant methamphetamine‐treated dams) were included to prevent the data from being biased by changes in gene expression that were overrepresented in a specific litter.

### 2.3 RNA sequencing

Library construction and RNA sequencing were performed by the Marshall University Genomics Core Facility. RNA libraries were prepared for each sample using the Truseq RNA mRNA library prep kit from Illumina. RNA sequencing (2 × 50 paired end reads; 20 million reads/sample) was performed on an Illumina HiSeq1500 sequencer.

RNA sequencing reads were aligned to the rat genome rn06 obtained from Ensembl, using HISAT2 version 2.1.0 (Kim et al., 2015), and the resulting BAM files were sorted using SAMtools version 1.9 (Li et al., 2009). Read quality was checked using FastQC (Andrews, 2010). Reads were trimmed to remove low‐confidence base calls and adapter sequences using Trimmomatic version 0.38 (Bolger et al., 2014), and then mapped to known transcripts from Ensembl genes version 94 using the R/Bioconductor package Genomic Alignments version 1.16.0 (Lawrence et al., 2013). Differentially expressed genes were identified using DESeq2 version 1.20.020 with false discovery rate (Benjamini–Hochberg adjusted *p*‐value) <0.1 used as a threshold for statistical significance (Benjamini & Hochberg, 1995).

### 2.4 Western blots

Protein expression for the genes, *DDAH2* and *BDH1*, was chosen since these genes were among the significant genes that exhibited the largest changes in expression and had *p*‐adjusted values less than *p* < 0.001 (Table S1). Frozen left ventricular tissue was homogenized with a Polytron in homogenization buffer [50 mM Tris, pH 7.4; 1 mM EDTA; 1% sodium dodecyl sulfate; phosphatase inhibitor cocktail 2 (catalog no. P5726, Sigma)]; phosphatase inhibitor cocktail 3 (catalog no. P0044, Sigma); and protease inhibitor cocktail (catalog no. P8340, Sigma) and immediately boiled for 5 min. Homogenates were centrifuged at 4°C for 10 min at 18 407 *g*. Supernatant protein (30 μg) was separated on a 10% polyacrylamide electrophoresis gel and subsequently transferred to a nitrocellulose membrane. The membrane was blocked with 5% nonfat dry milk and then blotted overnight with antibodies for dimethylarginine dimethlyaminohydrolase (DDAH2) (Proteintech #15417‐1‐AP; Rosemont, IL), 3‐betahydroxybutyrate dehydrogenase 1 (BDH1) (Abcam #ab184166; Cambridge, UK) or glyceraldehyde‐3‐phosphate dehydrogenase (GAPDH) (Cell Signaling, Danvers, MA; Catalog # 2118) prior to 1 h incubation with a horseradish peroxidase‐conjugated anti‐rabbit secondary antibody (Cell Signaling Technology # 7074). All primary antibodies were diluted 1 /1000 and the secondary antibodies were diluted 1/2000. Western blots were quantified by measurement of band densities with ImageJ software. Band intensities of DDAH2 and BDH1 were normalized to those of GAPDH.

### 2.5 Statistical analysis

Differentially expressed genes in RNA sequencing experiments were identified using DESeq2 version 1.20.0 (Love et al., 2014) with a false discovery rate (Benjamini–Hochberg adjusted *p*‐value) (Benjamini & Hochberg, 1995) less than FDR <0.1 used as a threshold for statistical significance. Statistical analyses of RNA sequencing data were limited to comparisons between hearts from saline and methamphetamine‐treated animals of the same sex. A sample size of 6 replicates per biological condition and a sequencing depth of 20 million reads per sample was chosen to optimize statistical power. Western blots were analyzed by the student's *t*‐test.

## 3 RESULTS

**FIGURE 1:**
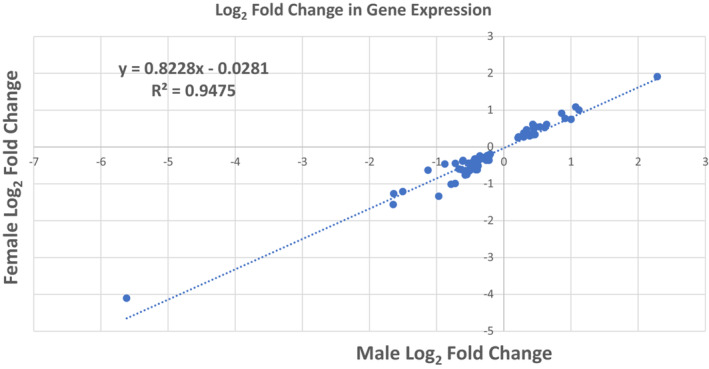
Plot of Log_2_ fold change for genes in both males (Saline *N* = 6; Methamphetamine *N* = 5) and females (Saline *N* = 5; Methamphetamine *N* = 6) exhibiting significant changes (FDR <0.01) in expression following prenatal exposure to methamphetamine.

**FIGURE 2:**
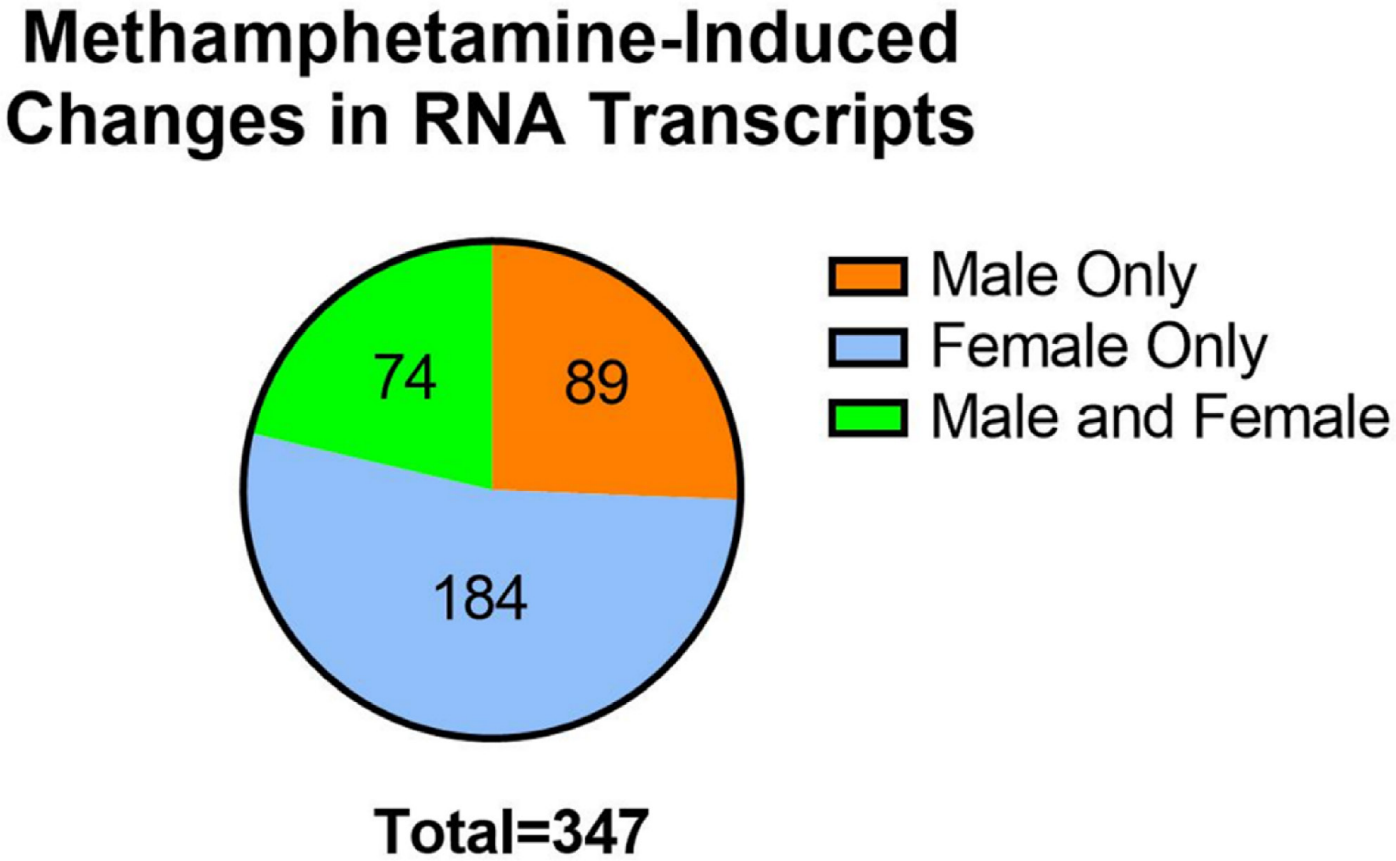
Distribution of genes between males (Saline *N* = 6; Methamphetamine *N* = 5) and females (Saline *N* = 5; Methamphetamine *N* = 6) exhibiting significant changes (FDR <0.01) in expression following prenatal exposure to methamphetamine.

Total RNA isolation from hearts of adult offspring prenatally exposed to methamphetamine and saline yielded RNA integrity numbers greater than or equal to 8.5 for all samples. Data from one female rat that had been prenatally exposed to methamphetamine was determined to be an outlier based on principal component analysis (Figure S1) and was excluded from further analyses. RNA sequencing identified 346 differentially (FDR <0.01) transcribed mRNAs in adult hearts that resulted from prenatal methamphetamine exposure (Tables S1–S3). Of the 346 differentially expressed transcripts identified, 74 methamphetamine‐induced changes were common to both males and females and showed similar patterns of expression both in magnitude and direction in both sexes (Figure 1 and Table S1). Prenatal methamphetamine induced changes in an additional 184 genes that occurred only in females (Table S2) and 89 changes that occurred only in males (Figure 2 and Table S3).

**FIGURE 3:**
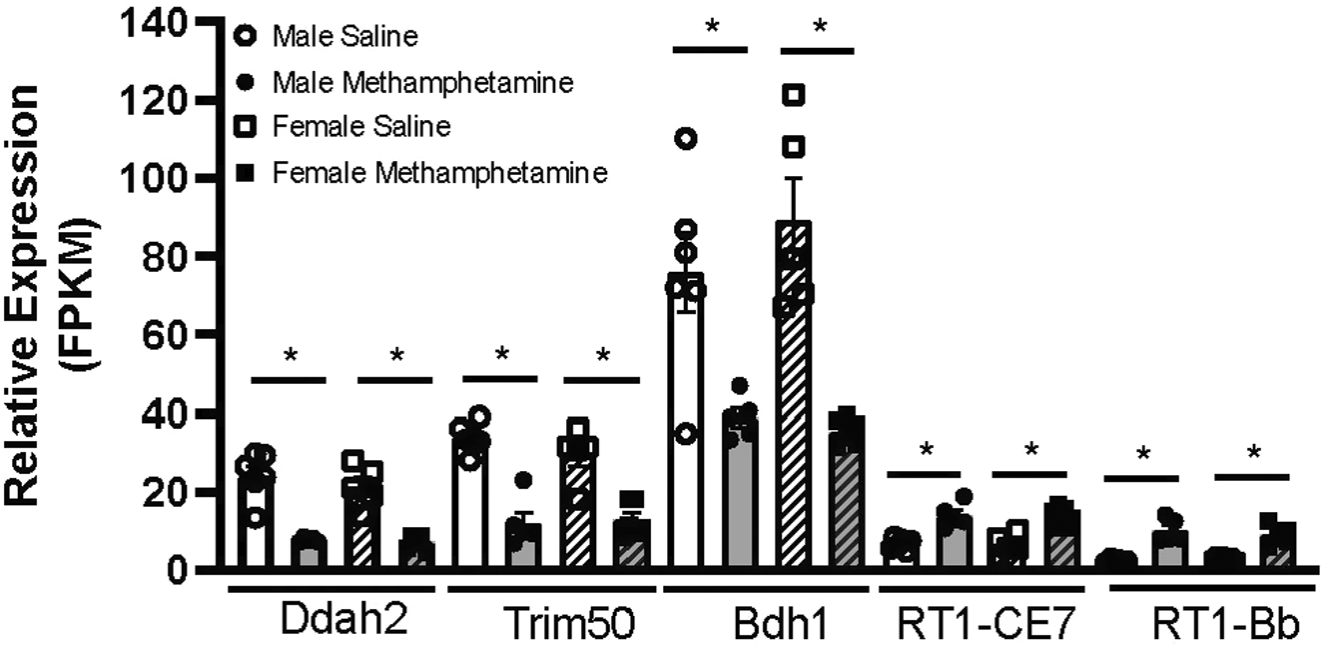
Relative expression of genes common to both males (Saline *N* = 6; Methamphetamine *N* = 5) and females (Saline *N* = 5; Methamphetamine *N* = 6). Genes significant (FDR <0.01) with Log_2_ fold change greater than 2. FPKM—fragments per kilobase per million reads sequenced, normalized both for the size of the transcript (in kilobases) and for the total number of reads sequenced for the sample. These values are comparable both across samples and across genes. Male Saline—open bar; Male Meth—closed bar; Female Saline—Open hatching; Female Meth—closed hatching.

A subset of these genes meeting higher stringency (FDR <0.01 and Log_2_ increase or decrease >1) were investigated for possible relevance to the observed phenotype of worsening ischemic injury to the heart during ischemia reperfusion experiments. The subset consisted of a total of 16 transcripts. Five transcripts were common to both males and females (Table 1). Interestingly, among significant transcripts expressed specifically in females at levels > 2‐fold none exhibited increased expression and of the genes specific to males only two showed increased expression (Table 1; Figure 3).

**TABLE 1:** List of significant (FDR < 0.01) genes with Log_2_ fold change >2

			Male			Female		
Down regulated—Both males and females			FoldChange (log_2_)	padj	effect	FoldChange (log_2_)	padj	effect
ENSRNOG00000000842	Dimethylarginine Dimethylaminohydrolase 2	Ddah2	−1.646	3.15 E–14	3.13	−1.561	4.19 E–13	2.95
ENSRNOG00000022483	Tripartite Motif Containing 50	Trim50	−1.504	1.37 E‐05	2.84	−1.210	1.65 E‐03	2.31
ENSRNOG00000001736	3‐Hydroxybutyrate Dehydrogenase 1	Bdh1	−0.968	1.34 E‐03	1.96	−1.337	2.19 E‐07	2.53
Up regulated—Both males and females								
ENSRNOG00000032708	RT1 class II, locus Bb	RT1‐Bb	2.286	2.39 E‐27	4.88	1.910	3.14 E‐18	3.76
ENSRNOG00000031090	RT1 class I, locus CE7	RT1‐CE7	1.071	2.44 E‐05	2.10	1.085	2.00 E‐05	2.12

Down regulated—Females only		Gene Symbol	FoldChange (Log_2_)	padj	effect
ENSRNOG00000020951	Solute carrier family 4 member 1	Slc4a1	−3.191	6.14 E‐17	9.13
ENSRNOG00000049766	Secretin receptor	Sctr	−1.022	9.59 E‐03	2.03
ENSRNOG00000037206	Coiled‐coil domain containing 77	Ccdc77	−1.012	2.05 E‐03	2.02

Down regulated—Males only		Gene Symbol	FoldChange (Log_2_)	padj	effect
ENSRNOG00000048951	RT1 class I, locus CE15	RT1‐CE15	−1.128	2.67 E‐05	2.19
ENSRNOG00000020837	Cd300 molecule‐like family member G	Cd300lg	−1.353	2.33 E‐04	2.55
ENSRNOG00000012067	FAM111 Trypsin Like Peptidase A	Fam111a	−5.616	5.34 E‐04	49.06
ENSRNOG00000013973	Lipocalin 2	Lcn2	−1.061	3.59 E‐03	2.09
ENSRNOG00000043451	Secreted phosphoprotein 1	Spp1	−3.358	4.38 E‐03	10.25
ENSRNOG00000016945	Phospholipase A2 Group IIA	Pla2g2a	−1.639	6.29 E‐03	3.11
UP regulated—Males only					
ENSRNOG00000014879	Tetratricopeptide repeat domain 7A	Ttc7a	1.002	3.45 E‐09	2.00
ENSRNOG00000038999	RT1 class Ia, locus A1	RT1‐A1	1.119	3.91 E‐03	2.17

*Note*: for Females only, there were no upregulated genes with a Log_2_ fold change greater than 2. Sample N's: males (Saline *N* = 6; Methamphetamine *N* = 5) and females (Saline *N* = 5; Methamphetamine *N* = 6).

**FIGURE 4:**
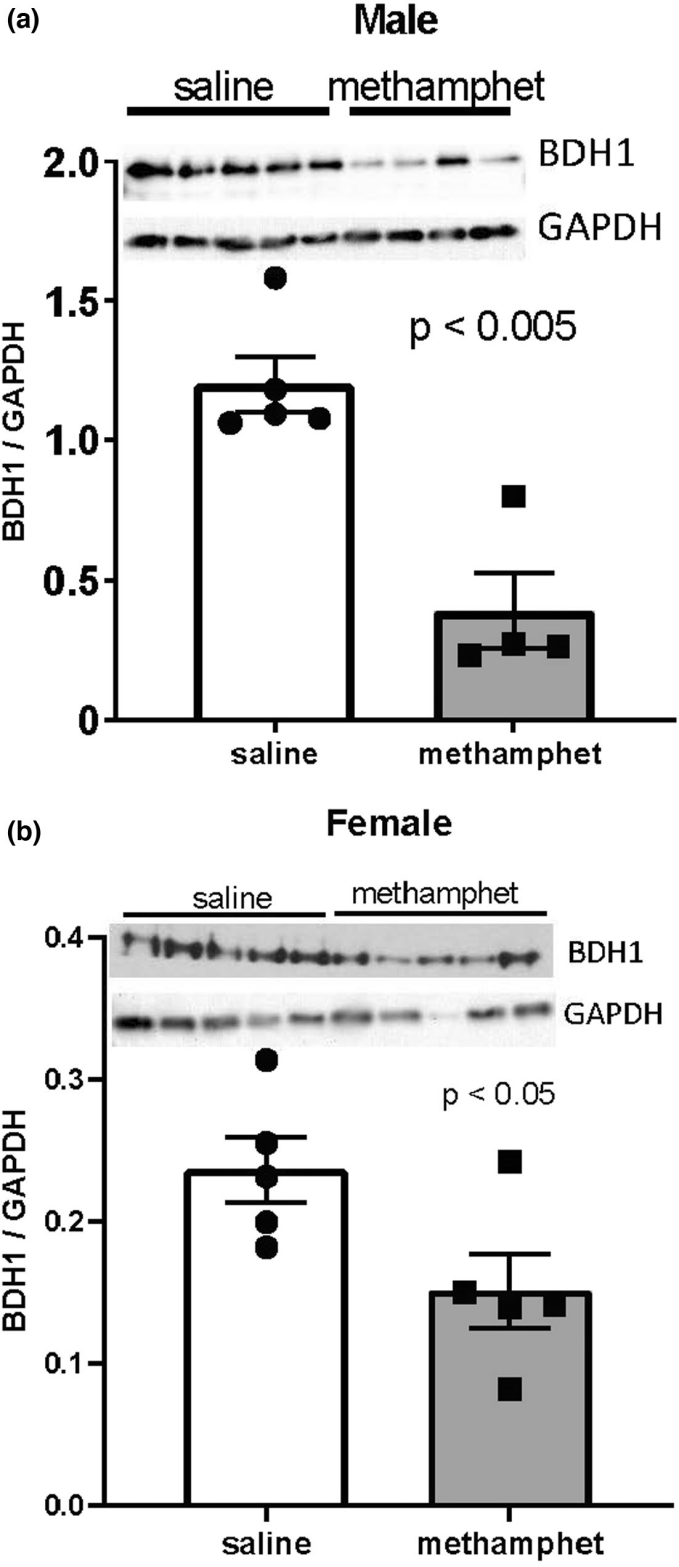
Expression of 3‐hydroxybutyrate dehydrogenase (BDH1)1 in hearts of offspring measured by western blotting. (a) DDAH2 expression in adult male ventricles. (b) DDAH2 expression in adult female ventricles. Error bars denote Standard Errors.

**FIGURE 5:**
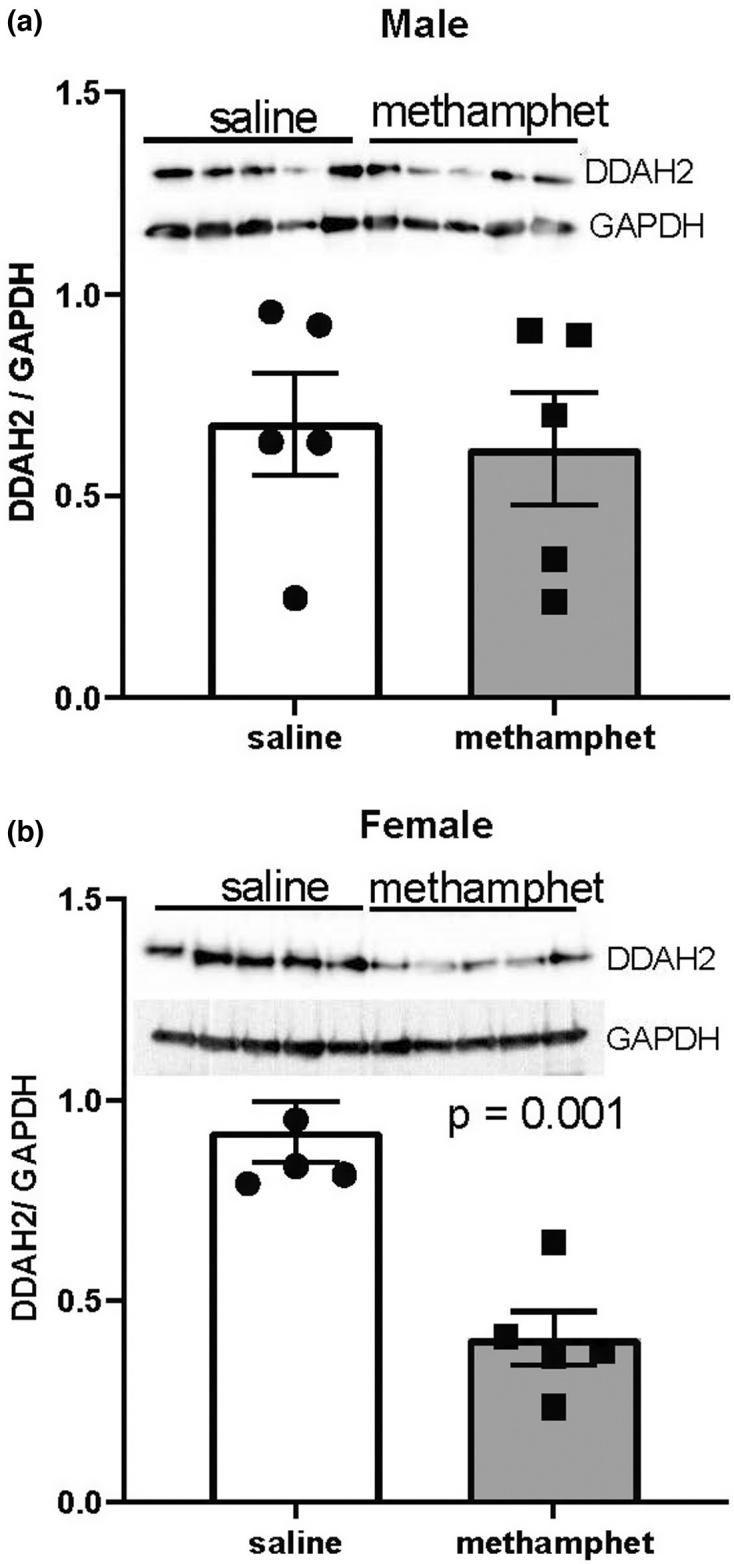
Expression of dimethylarginine dimethylaminohydrolase 2 (DDAH2) in hearts of offspring measured by western blotting. (a) DDAH2 expression in adult male ventricles. (b) DDAH2 expression in adult female ventricles. Error bars denote Standard Errors.

Dimethylargenine dimethylaminohydrolase‐2 (DDAH2) and 3‐hydroxybutyrate dehydrogenase 1 (BDH1) were among the largest changes in gene expression that were common to both male and female hearts. Importantly, the proteins encoded by these genes regulate metabolic pathways that are involved in heart failure. Western blotting indicated that 3‐hydroxybutyrate dehydrogenase 1 (BDH1) expression was significantly decreased at the protein level in hearts from both male (*p* < 0.01) and female (*p* < 0.05) offspring that had been prenatally exposed to methamphetamine (Figure 4). Dimethylarginine dimethylaminohydrolase 2 (DDAH2) expression was significantly decreased in female hearts (*p* < 0.01) but was not significantly altered by prenatal exposure to methamphetamine in adult male hearts (Figure 5).

## 4 DISCUSSION

The behavioral, neurological, and cognitive impacts of prenatal exposure to methamphetamine have been well studied (Chang et al., 2004; Diaz et al., 2014; LaGasse et al., 2012; Roos et al., 2014). Recent studies have demonstrated that fetal exposure to methamphetamine induces epigenetic changes in gene expression that can result in long‐term phenotypic effects in the adult offspring. For example, fetal exposure to methamphetamine alters gene expression and DNA methylation patterns in the brain of adult offspring (Dong et al., 2018; Itzhak et al., 2015). In addition, Korchynska et al. reported that prenatal exposure to methamphetamine disrupts the expression of pancreatic genes required for insulin production. This results in life‐long disruption of glucose homeostasis in adult offspring (Korchynska et al., 2020). The present study is the first investigation that we are aware of to assess prenatal methamphetamine‐induced changes in mRNA transcripts in the hearts of adult offspring. These data demonstrate that the female heart is more susceptible, in terms of the number of genes significantly altered, than the male heart to methamphetamine‐induced changes in the cardiac transcriptome that occur during the gestational period. This is consistent with our previous work demonstrating that prenatal exposure to methamphetamine selectively sensitizes the adult female heart to ischemic injury (Rorabaugh et al., 2016). Collectively, these data suggest that methamphetamine use during pregnancy may increase the risk of cardiac disorders in the adult offspring.

Transcripts encoding 3‐hydroxybutyrate dehydrogenase 1 (*BDH1*) and dimethylarginine dimethylaminohydrolase 2 (*DDAH2*) were among the most significantly changed genes in both male and female hearts (Table 1). These genes were selected for further analysis by Western blotting because of their involvement in metabolic pathways related to heart failure. Western blotting indicated that BDH1 expression was significantly decreased at the protein level in both male and female hearts following prenatal exposure to methamphetamine (Figure 4). Bdh1 catalyzes the conversion of betahydroxybutyrate to acetoacetate which is subsequently converted to acetyl CoA and fed into the Krebs cycle where it is used to generate ATP (Abdul Kadir et al., 2020). Under normal conditions, ketone bodies account for only a small fraction of myocardial ATP production (Abdul Kadir et al., 2020). However, cardiac hypertrophy induces a metabolic shift that causes the heart to become more dependent on ketone bodies to meet its metabolic needs (Aubert et al., 2016; Bedi Jr. et al., 2016; Voros et al., 2018). Horton et al. (2019) found that cardiac function (ejection fraction, end systolic and diastolic volumes) was significantly worsened in cardiac‐specific Bdh1 knockout mice compared with wildtype animals following transverse aortic constriction (TAC)‐induced pressure overload (Horton et al., 2019). Consistent with this finding, cardiac specific overexpression of Bdh1 attenuated cardiac dysfunction and remodeling in heart failure (Uchihashi et al., 2017). These previous studies provide compelling evidence that Bdh1 expression plays a key role in cardiac contractile dysfunction and structural remodeling during heart failure, but it is unknown whether methamphetamine‐induced inhibition of Bdh1 compromises the heart's ability to utilize ketones, worsens cardiac dysfunction, or worsens structural remodeling of the heart under conditions of heart failure.

Western blotting indicated that DDAH2 expression was significantly decreased at the protein level in female hearts, but not in male hearts following prenatal exposure to methamphetamine (Figure 5) despite both male and female offspring showing significant decreases in *DDAH2* gene expression suggesting some compensatory mechanism in males. DDAH2 metabolizes asymmetric dimethylarginine (ADMA), an endogenous inhibitor of all three isoforms of nitric oxide synthase (Leiper et al., 2007). Suppression of DDAH2 expression results in the accumulation of ADMA and subsequent inhibition of nitric oxide‐dependent signaling (Böger, 2003; Leiper et al., 2007; Palm et al., 2007). Cardiac expression of DDAH2 is decreased in canine (Chen et al., 2005) and rodent (Zhou et al., 2017) models of heart failure, and the resulting increase in ADMA concentration has been implicated in endothelial dysfunction that accompanies heart failure (Böger, 2003, 2004; Liu et al., 2018). In addition, elevated serum ADMA concentrations are predictive of poor outcomes in people that have heart failure (Hsu et al., 2012; Saitoh et al., 2003; Usui et al., 1998). Hearts from rats that were prenatally exposed methamphetamine did not display any overt signs of contractile dysfunction or hypertrophy either in this study or in our prior work (Rorabaugh et al., 2016). However, our finding that Bdh1 and DDAH2 expression are decreased following prenatal exposure to methamphetamine suggests that individuals that develop heart failure as adults may experience more severe contractile dysfunction and cardiac remodeling if they were prenatally exposed to methamphetamine (Chavva & Rorabaugh, 2022; Rorabaugh et al., 2016).

In addition to our studies of the cardiac effects prenatal exposure to methamphetamine, we have also investigated the cardiac impact of methamphetamine exposure during early adulthood. We previously reported that adult female (but not male) rats that were treated with methamphetamine for 10 consecutive days developed myocardial hypersensitivity to ischemia (Rorabaugh et al., 2017). This sex‐dependent cardiac phenotype was similar to that observed following prenatal exposure to methamphetamine (Rorabaugh et al., 2016, 2017). RNA sequencing of hearts from these animals identified 340 changes in gene expression 24 h after the final methamphetamine injection (Chavva et al., 2021). Similar to the present study, the vast majority (283 out of 340; 83%) of these changes in cardiac gene expression occurred exclusively in female hearts. Furthermore, the magnitude of the changes was significantly greater in female hearts than male hearts when the animals were exposed to methamphetamine during early adulthood (Chavva et al., 2021). Thus, the female heart is more susceptible to changes in cardiac gene expression regardless of whether methamphetamine exposure occurs during the prenatal period or during early adulthood. However, comparison of methamphetamine induced changes during the prenatal period and during early adulthood revealed no similarities in the identity of the genes that were changed. Notably, 5 of the top 6 changes in gene expression (in terms of the magnitude of the changes) in hearts from females that were exposed to methamphetamine as adults involved genes that regulate the circadian clock (*Dbp, Per2, Per3, Arntl*, and *Npas2*) (Chavva et al., 2022). These genes were not altered in hearts of adult rats following prenatal exposure to methamphetamine. Likewise, prenatal methamphetamine exposure in the present study induced changes in proteins that have been implicated in heart failure (DDAH2 and Bdh1) and function of the major histocompatibility complex (RT‐1Bb, RT1‐CE7, RT1‐CE15, RT1‐A1) but were not significantly impacted when methamphetamine was administered during adulthood (Chavva et al., 2021). Thus, exposure to methamphetamine during either the prenatal period or during early adulthood both results in a female specific phenotype that is characterized by myocardial hypersensitivity to ischemic injury changes in cardiac gene expression that occur predominately in female hearts. However, the specific genes that are altered by prenatal or adult exposure to methamphetamine are markedly different.

Other investigators have reported that prenatal exposure to methamphetamine induces changes in gene expression in the brain and pancreas that are mediated by epigenetic mechanisms such as DNA methylation. Korchynska et al. (2020) found that prenatal exposure to methamphetamine caused hypermethylation and decreased expression of genes required for insulin production in the pancreas of adult mice. These animals displayed permanent impairment of insulin secretion and glucose homeostasis. Fetal exposure to methamphetamine also results in DNA methylation and changes in the expression of neurodevelopmental genes in the hippocampus and nucleus accumbens of the brain, resulting in impaired memory (Dong et al., 2018) and reduced response to conditioned fear (Itzhak et al., 2015). Thus, the finding that prenatal exposure to methamphetamine leads to changes in myocardial gene expression in adult offspring is consistent with previous findings in other organs.

The impact of prenatal methamphetamine exposure on the cardiovascular system of adult offspring is not limited to the heart. We recently reported that chronic fetal exposure to methamphetamine leads to vascular dysfunction in adult offspring. Adult (5 months old) rats that were exposed to methamphetamine throughout the gestational period exhibited vascular dysfunction characterized by potentiation of angiotensin II‐induced vasoconstriction and attenuation of perivascular adipose tissue (PVAT)‐dependent relaxation (Chavva et al., 2022). In contrast to the effects of prenatal methamphetamine on the ischemic heart (Rorabaugh et al., 2016), the effects of prenatal methamphetamine on vascular function occurred exclusively in male offspring. Thus, our data provide evidence that prenatal exposure to methamphetamine differentially alters both cardiac and vascular function in a sex‐dependent manner. Our findings suggest that individuals that were prenatally exposed to methamphetamine may be at increased risk of developing cardiovascular disorders during adulthood.

In summary, our data provide evidence that prenatal exposure to methamphetamine induces changes in myocardial gene expression that persist into adulthood. Furthermore, many of these changes are sex‐dependent with the majority occurring exclusively in female hearts. Further work is needed to determine whether these changes ultimately lead to adult‐onset cardiovascular disorders later in life.

## 5 CONCLUSION

These results indicate that methamphetamine exposure during gestation causes gene and protein expression changes in myocardium that persist into adulthood. These changes occur in both sex‐dependent and sex independent manners. DDAH2 expression is decreased only in females while BDH1 expression is decreased in males and females. These results are consistent with evidence of worsened cardiac injury from ischemia following disruptions to the NO synthase pathway. RNA sequencing also showed that gene transcription was altered more significantly in females alone than females and males or males alone. These results are consistent with past research showing females are more susceptible to cardiac alterations and damage following prenatal methamphetamine exposure. Further research is needed to determine whether changes in the expression of DDAH2 or BDH1 underlie worsening of cardiac ischemic injury in adult female offspring following prenatal exposure to methamphetamine.

## AUTHOR CONTRIBUTIONS

HC performed daily injections and identified pregnant rats. HC and BR performed surgeries. HC and DB performed RNA isolations. JD performed RNA sequencing. AD and BR identified proteins of interest. HC, AD, and BR performed Western blotting. BR designed the experiments and analyzed the data. All authors have read and approved the manuscript.

## FUNDING INFORMATION

This work was funded by Supported by the National Heart Lung Blood Institute [R15HL145546]. Support was also provided by the American Heart Association [18UFEL33960330], the WV‐. INBRE grant [P20GM103434], the COBRE ACCORD grant [1P20GM121299], and the West Virginia Clinical and Translational Science Institute (WV‐CTSI) grant [2U54GM104942].

## CONFLICT OF INTEREST

No conflicts of interest, financial, or otherwise are declared by the authors.

## ETHICS STATEMENT

The manuscript contains no data or description of human patients. All procedures involving animals were approved by the Institutional Animal Care and Use Committee of Marshall University.

## Supporting information


Appendix S1
