# Supplementary material for: Maternal use of methamphetamine induces sex‐dependent changes in myocardial gene expression in adult offspring
Source: Physiol Rep. 2022 Nov 25;10(22):e15509. doi: 10.14814/phy2.15509 (PMC9693808; doi:10.14814/phy2.15509)
Supplement: Supplementary file 1 — Appendix S1 [file PHY2-10-e15509-s001.docx]

Table S1. Significant genes (FDR<0.01) common to both Males and Females.

| **Down Regulated** | | **Male** | | | **Female** | | |
| --- | --- | --- | --- | --- | --- | --- | --- |
|  | Gene Symbol | FoldChange (log_2_) | padj | effect | FoldChange (log_2_) | padj | effect |
| ENSRNOG00000012067 | Fam111a | -5.616 | 5.34E-04 | 49.06 | -4.104 | 3.29E-02 | 17.19 |
| ENSRNOG00000000842 | Ddah2 | -1.646 | 3.15E-14 | 3.13 | -1.561 | 4.19E-13 | 2.95 |
| ENSRNOG00000016945 | Pla2g2a | -1.639 | 6.29E-03 | 3.11 | -1.267 | 6.55E-02 | 2.41 |
| ENSRNOG00000022483 | Trim50 | -1.504 | 1.37E-05 | 2.84 | -1.210 | 1.65E-03 | 2.31 |
| ENSRNOG00000048951 | RT1-CE15 | -1.128 | 2.67E-05 | 2.19 | -0.629 | 9.32E-02 | 1.55 |
| ENSRNOG00000001736 | Bdh1 | -0.968 | 1.34E-03 | 1.96 | -1.337 | 2.19E-07 | 2.53 |
| ENSRNOG00000018910 | Abhd11 | -0.877 | 2.42E-06 | 1.84 | -0.461 | 6.32E-02 | 1.38 |
| ENSRNOG00000037206 | Ccdc77 | -0.784 | 5.00E-02 | 1.72 | -1.012 | 2.05E-03 | 2.02 |
| ENSRNOG00000020251 | Art1 | -0.723 | 6.42E-02 | 1.65 | -0.991 | 1.49E-03 | 1.99 |
| ENSRNOG00000000456 | Psmb8 | -0.721 | 1.27E-04 | 1.65 | -0.444 | 8.30E-02 | 1.36 |
| ENSRNOG00000017286 | Ephx2 | -0.666 | 2.93E-11 | 1.59 | -0.598 | 1.17E-09 | 1.51 |
| ENSRNOG00000000473 | Pfdn6 | -0.628 | 6.33E-07 | 1.55 | -0.615 | 9.15E-07 | 1.53 |
| ENSRNOG00000049071 | Phyhip | -0.613 | 1.37E-05 | 1.53 | -0.384 | 3.26E-02 | 1.31 |
| ENSRNOG00000010555 | Phyhip | -0.607 | 1.09E-05 | 1.52 | -0.365 | 4.05E-02 | 1.29 |
| ENSRNOG00000017895 | Eno1 | -0.579 | 3.97E-10 | 1.49 | -0.656 | 1.68E-13 | 1.58 |
| ENSRNOG00000009192 | Rnaseh2b | -0.574 | 3.77E-02 | 1.49 | -0.761 | 1.43E-03 | 1.70 |
| ENSRNOG00000021248 | Cdc25b | -0.563 | 6.45E-02 | 1.48 | -0.640 | 1.27E-02 | 1.56 |
| ENSRNOG00000000281 | Prodh1 | -0.550 | 4.45E-02 | 1.46 | -0.749 | 5.26E-04 | 1.68 |
| ENSRNOG00000049269 | NA | -0.527 | 7.29E-11 | 1.44 | -0.649 | 3.44E-17 | 1.57 |
| ENSRNOG00000001232 | Slc19a1 | -0.527 | 2.54E-02 | 1.44 | -0.440 | 7.61E-02 | 1.36 |
| ENSRNOG00000028543 | NA | -0.512 | 5.48E-10 | 1.43 | -0.649 | 7.56E-17 | 1.57 |
| ENSRNOG00000012480 | Pxylp1 | -0.493 | 4.31E-02 | 1.41 | -0.618 | 2.09E-03 | 1.53 |
| ENSRNOG00000028746 | Gsto1 | -0.490 | 2.54E-03 | 1.40 | -0.556 | 2.94E-04 | 1.47 |
| ENSRNOG00000017843 | Polr3k | -0.448 | 2.54E-03 | 1.36 | -0.382 | 1.72E-02 | 1.30 |
| ENSRNOG00000049056 | NA | -0.442 | 8.70E-11 | 1.36 | -0.464 | 4.08E-12 | 1.38 |
| ENSRNOG00000018736 | Pnpla2 | -0.435 | 2.89E-05 | 1.35 | -0.354 | 2.09E-03 | 1.28 |
| ENSRNOG00000043192 | Hacd1 | -0.431 | 8.32E-03 | 1.35 | -0.324 | 9.32E-02 | 1.25 |
| ENSRNOG00000005195 | Cst3 | -0.430 | 2.70E-05 | 1.35 | -0.402 | 1.40E-04 | 1.32 |
| ENSRNOG00000015239 | Ginm1 | -0.429 | 6.74E-05 | 1.35 | -0.332 | 7.63E-03 | 1.26 |
| ENSRNOG00000047551 | LOC100911615 | -0.422 | 1.37E-05 | 1.34 | -0.322 | 3.22E-03 | 1.25 |
| ENSRNOG00000024763 | NA | -0.417 | 7.96E-06 | 1.34 | -0.607 | 1.36E-13 | 1.52 |
| ENSRNOG00000012658 | Pdlim3 | -0.416 | 5.34E-04 | 1.33 | -0.617 | 5.32E-10 | 1.53 |
| ENSRNOG00000012684 | Bloc1s2 | -0.411 | 4.76E-02 | 1.33 | -0.586 | 2.92E-04 | 1.50 |
| ENSRNOG00000021338 | Tmem132a | -0.406 | 1.47E-02 | 1.33 | -0.376 | 2.51E-02 | 1.30 |
| ENSRNOG00000049385 | Adamtsl4 | -0.402 | 2.54E-03 | 1.32 | -0.373 | 4.60E-03 | 1.30 |
| ENSRNOG00000062283 | NA | -0.395 | 1.58E-03 | 1.32 | -0.617 | 1.17E-09 | 1.53 |
| ENSRNOG00000001229 | Col18a1 | -0.391 | 1.03E-02 | 1.31 | -0.416 | 5.25E-03 | 1.34 |
| ENSRNOG00000020607 | Bckdha | -0.388 | 1.51E-02 | 1.31 | -0.351 | 3.29E-02 | 1.28 |
| ENSRNOG00000028993 | LOC102555453 | -0.382 | 2.76E-03 | 1.30 | -0.512 | 2.81E-06 | 1.43 |
| ENSRNOG00000020994 | Slc25a39 | -0.356 | 2.29E-04 | 1.28 | -0.240 | 4.45E-02 | 1.18 |
| ENSRNOG00000001827 | Masp1 | -0.341 | 5.10E-02 | 1.27 | -0.323 | 5.89E-02 | 1.25 |
| ENSRNOG00000016827 | Slc38a3 | -0.284 | 7.86E-02 | 1.22 | -0.279 | 5.89E-02 | 1.21 |
| ENSRNOG00000029115 | LOC102555453 | -0.261 | 7.03E-02 | 1.20 | -0.359 | 1.53E-03 | 1.28 |
| ENSRNOG00000033748 | NA | -0.249 | 5.90E-02 | 1.19 | -0.358 | 4.17E-04 | 1.28 |
| ENSRNOG00000000503 | Ppard | -0.247 | 8.92E-02 | 1.19 | -0.233 | 9.32E-02 | 1.18 |
| ENSRNOG00000013201 | Tex264 | -0.240 | 8.13E-02 | 1.18 | -0.243 | 5.66E-02 | 1.18 |
| ENSRNOG00000061579 | NA | -0.220 | 8.57E-02 | 1.16 | -0.360 | 6.73E-05 | 1.28 |
| ENSRNOG00000016532 | Ino80c | -0.218 | 7.87E-02 | 1.16 | -0.251 | 1.99E-02 | 1.19 |
| ENSRNOG00000012406 | Pcbp4 | -0.217 | 6.01E-02 | 1.16 | -0.211 | 5.70E-02 | 1.16 |
| ENSRNOG00000025443 | Map1lc3a | -0.200 | 5.10E-02 | 1.15 | -0.194 | 5.12E-02 | 1.14 |
|  |  |  |  |  |  |  |  |
| **Up Regulated** | | **Male** | | | **Female** | | |
|  | Gene Symbol | FoldChange (log_2_) | padj | effect | FoldChange (log_2_) | padj | effect |
| ENSRNOG00000032708 | RT1-Bb | 2.286 | 2.39E-27 | 4.88 | 1.910 | 3.14E-18 | 3.76 |
| ENSRNOG00000031090 | RT1-CE7 | 1.071 | 2.44E-05 | 2.10 | 1.085 | 2.00E-05 | 2.12 |
| ENSRNOG00000038999 | RT1-A1 | 1.119 | 3.91E-03 | 2.17 | 1.004 | 1.52E-02 | 2.01 |
| ENSRNOG00000001726 | Tmem44 | 0.862 | 1.09E-05 | 1.82 | 0.914 | 5.59E-06 | 1.88 |
| ENSRNOG00000006108 | Gngt2 | 0.914 | 1.08E-03 | 1.88 | 0.777 | 1.91E-02 | 1.71 |
| ENSRNOG00000014879 | Ttc7a | 1.002 | 3.45E-09 | 2.00 | 0.750 | 7.75E-05 | 1.68 |
| ENSRNOG00000015124 | Gpam | 0.432 | 2.07E-03 | 1.35 | 0.614 | 2.16E-07 | 1.53 |
| ENSRNOG00000015420 | Stxbp1 | 0.638 | 6.33E-07 | 1.56 | 0.613 | 2.49E-06 | 1.53 |
| ENSRNOG00000032798 | Slco3a1 | 0.461 | 1.55E-02 | 1.38 | 0.560 | 8.03E-04 | 1.47 |
| ENSRNOG00000024924 | Cep19 | 0.440 | 4.82E-02 | 1.36 | 0.552 | 3.12E-03 | 1.47 |
| ENSRNOG00000056944 | Arhgap24 | 0.536 | 1.27E-02 | 1.45 | 0.548 | 7.81E-03 | 1.46 |
| ENSRNOG00000007610 | Gdf11 | 0.610 | 3.03E-03 | 1.53 | 0.528 | 2.52E-02 | 1.44 |
| ENSRNOG00000052687 | Megf8 | 0.449 | 9.35E-02 | 1.37 | 0.492 | 3.82E-02 | 1.41 |
| ENSRNOG00000004812 | Sema6d | 0.339 | 9.22E-02 | 1.27 | 0.469 | 3.27E-03 | 1.38 |
| ENSRNOG00000015093 | Sparcl1 | 0.350 | 4.38E-03 | 1.28 | 0.382 | 1.10E-03 | 1.30 |
| ENSRNOG00000016968 | Gramd4 | 0.297 | 5.10E-02 | 1.23 | 0.379 | 3.22E-03 | 1.30 |
| ENSRNOG00000008082 | Rgs6 | 0.437 | 3.28E-03 | 1.35 | 0.364 | 2.52E-02 | 1.29 |
| ENSRNOG00000057817 | Epb41l1 | 0.465 | 1.20E-03 | 1.38 | 0.337 | 4.97E-02 | 1.26 |
| ENSRNOG00000055809 | LOC100911769 | 0.441 | 6.40E-03 | 1.36 | 0.333 | 8.34E-02 | 1.26 |
| ENSRNOG00000025895 | Cavin2 | 0.312 | 3.29E-02 | 1.24 | 0.323 | 1.99E-02 | 1.25 |
| ENSRNOG00000001762 | Pcyt1a | 0.386 | 1.38E-05 | 1.31 | 0.301 | 3.01E-03 | 1.23 |
| ENSRNOG00000057589 | Rnf11l1 | 0.221 | 6.97E-02 | 1.17 | 0.276 | 7.73E-03 | 1.21 |
| ENSRNOG00000018239 | Dhrs4 | 0.212 | 6.91E-02 | 1.16 | 0.248 | 1.54E-02 | 1.19 |

Table S2. Significant genes (FDR>0.01) for Females only.

| **Female Down Regulated** | | | | |
| --- | --- | --- | --- | --- |
|  | Gene Symbol | FoldChange (Log_2_) | padj | effect |
| ENSRNOG00000020951 | Slc4a1 | -3.191 | 6.14E-17 | 9.13 |
| ENSRNOG00000049766 | Sctr | -1.022 | 9.59E-03 | 2.03 |
| ENSRNOG00000008943 | Penk | -1.018 | 8.27E-02 | 2.03 |
| ENSRNOG00000017560 | Mdk | -0.897 | 2.46E-02 | 1.86 |
| ENSRNOG00000018285 | Kcna2 | -0.715 | 5.12E-02 | 1.64 |
| ENSRNOG00000061910 | Igfbp3 | -0.690 | 6.03E-03 | 1.61 |
| ENSRNOG00000012660 | Postn | -0.647 | 3.55E-02 | 1.57 |
| ENSRNOG00000042825 | Cd300le | -0.636 | 4.19E-02 | 1.55 |
| ENSRNOG00000046700 | Mettl27 | -0.606 | 3.85E-02 | 1.52 |
| ENSRNOG00000033625 | NA | -0.587 | 8.26E-02 | 1.50 |
| ENSRNOG00000017328 | Pter | -0.568 | 6.83E-02 | 1.48 |
| ENSRNOG00000015077 | Acsf3 | -0.567 | 1.43E-03 | 1.48 |
| ENSRNOG00000021145 | Gpr137 | -0.566 | 9.15E-07 | 1.48 |
| ENSRNOG00000053201 | Gpcpd1 | -0.565 | 3.03E-03 | 1.48 |
| ENSRNOG00000033215 | RT1-Db1 | -0.564 | 5.00E-02 | 1.48 |
| ENSRNOG00000010799 | Noct | -0.555 | 8.67E-02 | 1.47 |
| ENSRNOG00000013128 | Tmem179 | -0.549 | 6.71E-03 | 1.46 |
| ENSRNOG00000032364 | Tbcel | -0.533 | 6.29E-02 | 1.45 |
| ENSRNOG00000052415 | Gstt2 | -0.515 | 1.04E-02 | 1.43 |
| ENSRNOG00000015382 | Arid5a | -0.513 | 5.81E-03 | 1.43 |
| ENSRNOG00000018649 | Slc35c2 | -0.490 | 5.81E-03 | 1.40 |
| ENSRNOG00000020659 | Mrpl4 | -0.477 | 5.45E-05 | 1.39 |
| ENSRNOG00000048725 | Lsm2 | -0.473 | 7.32E-02 | 1.39 |
| ENSRNOG00000007541 | Fhl3 | -0.472 | 7.81E-03 | 1.39 |
| ENSRNOG00000013097 | LOC691485 | -0.459 | 1.73E-02 | 1.37 |
| ENSRNOG00000007837 | Acot11 | -0.457 | 7.22E-02 | 1.37 |
| ENSRNOG00000005193 | Pfas | -0.454 | 8.78E-02 | 1.37 |
| ENSRNOG00000020729 | Stc2 | -0.452 | 3.55E-02 | 1.37 |
| ENSRNOG00000061519 | Asap2 | -0.444 | 4.64E-02 | 1.36 |
| ENSRNOG00000012582 | Eif4ebp1 | -0.441 | 5.89E-02 | 1.36 |
| ENSRNOG00000019689 | Vwf | -0.433 | 4.05E-02 | 1.35 |
| ENSRNOG00000050401 | Vbp1 | -0.428 | 2.14E-03 | 1.35 |
| ENSRNOG00000020635 | Exosc5 | -0.421 | 6.83E-02 | 1.34 |
| ENSRNOG00000008090 | Txndc12 | -0.403 | 3.01E-03 | 1.32 |
| ENSRNOG00000007896 | Klhl38 | -0.394 | 1.52E-02 | 1.31 |
| ENSRNOG00000000302 | Sesn1 | -0.390 | 2.55E-02 | 1.31 |
| ENSRNOG00000030871 | Calm2 | -0.377 | 6.04E-04 | 1.30 |
| ENSRNOG00000037627 | Trappc1 | -0.372 | 7.42E-02 | 1.29 |
| ENSRNOG00000003452 | Asb11 | -0.370 | 3.56E-02 | 1.29 |
| ENSRNOG00000062013 | Adprhl1 | -0.369 | 2.86E-02 | 1.29 |
| ENSRNOG00000025730 | Armcx3 | -0.364 | 7.42E-02 | 1.29 |
| ENSRNOG00000042274 | Fbxo31 | -0.358 | 5.70E-03 | 1.28 |
| ENSRNOG00000012960 | Uap1l1 | -0.356 | 1.63E-02 | 1.28 |
| ENSRNOG00000008510 | Abtb2 | -0.350 | 2.43E-02 | 1.28 |
| ENSRNOG00000007338 | Fbln2 | -0.349 | 7.17E-02 | 1.27 |
| ENSRNOG00000011603 | Cab39l | -0.343 | 9.45E-02 | 1.27 |
| ENSRNOG00000017382 | Snx33 | -0.339 | 2.46E-02 | 1.27 |
| ENSRNOG00000030106 | Rps10l1 | -0.332 | 7.24E-02 | 1.26 |
| ENSRNOG00000024885 | Asb10 | -0.329 | 3.95E-02 | 1.26 |
| ENSRNOG00000019232 | RGD1311345 | -0.324 | 3.35E-02 | 1.25 |
| ENSRNOG00000013660 | Cog6 | -0.315 | 3.82E-02 | 1.24 |
| ENSRNOG00000018033 | Ddx19a | -0.309 | 1.27E-02 | 1.24 |
| ENSRNOG00000008829 | Sorbs3 | -0.304 | 9.74E-02 | 1.23 |
| ENSRNOG00000049099 | NA | -0.301 | 3.30E-02 | 1.23 |
| ENSRNOG00000010593 | Ctnnal1 | -0.298 | 3.31E-02 | 1.23 |
| ENSRNOG00000018590 | Drg1 | -0.293 | 8.49E-02 | 1.23 |
| ENSRNOG00000002914 | Trmt1 | -0.288 | 8.56E-02 | 1.22 |
| ENSRNOG00000009745 | Ppp3cc | -0.286 | 1.13E-02 | 1.22 |
| ENSRNOG00000008079 | Ugp2 | -0.278 | 9.27E-03 | 1.21 |
| ENSRNOG00000002194 | Coq2 | -0.273 | 9.32E-02 | 1.21 |
| ENSRNOG00000016684 | Wnk2 | -0.270 | 1.65E-03 | 1.21 |
| ENSRNOG00000017188 | Cyp27a1 | -0.265 | 5.04E-02 | 1.20 |
| ENSRNOG00000048812 | Gpx1 | -0.257 | 4.36E-02 | 1.20 |
| ENSRNOG00000049484 | Atp9a | -0.243 | 8.30E-02 | 1.18 |
| ENSRNOG00000013346 | Asb14 | -0.242 | 4.38E-02 | 1.18 |
| ENSRNOG00000033517 | LOC100360791 | -0.241 | 8.48E-02 | 1.18 |
| ENSRNOG00000001222 | Ube2g2 | -0.237 | 7.42E-02 | 1.18 |
| ENSRNOG00000010170 | Tubb4b | -0.235 | 3.31E-02 | 1.18 |
| ENSRNOG00000017087 | Man1c1 | -0.231 | 4.50E-02 | 1.17 |
| ENSRNOG00000046763 | Adssl1 | -0.228 | 6.21E-02 | 1.17 |
| ENSRNOG00000055647 | Rbfa | -0.228 | 9.18E-02 | 1.17 |
| ENSRNOG00000009832 | Slc39a14 | -0.227 | 8.49E-02 | 1.17 |
| ENSRNOG00000011168 | Micu2 | -0.225 | 8.70E-02 | 1.17 |
| ENSRNOG00000018651 | Agtpbp1 | -0.224 | 9.44E-03 | 1.17 |
| ENSRNOG00000010838 | Araf | -0.222 | 4.47E-02 | 1.17 |
| ENSRNOG00000052141 | Rps10l1 | -0.217 | 8.19E-02 | 1.16 |
| ENSRNOG00000061348 | Fam53b | -0.213 | 5.89E-02 | 1.16 |
| ENSRNOG00000019742 | Stat3 | -0.212 | 4.05E-02 | 1.16 |
| ENSRNOG00000009244 | Abhd4 | -0.206 | 6.32E-02 | 1.15 |
| ENSRNOG00000013514 | Maf1 | -0.203 | 9.07E-02 | 1.15 |
| ENSRNOG00000020897 | Prpf19 | -0.199 | 6.83E-02 | 1.15 |
| ENSRNOG00000039980 | Slc25a5 | -0.198 | 2.46E-02 | 1.15 |
| ENSRNOG00000011489 | R3hdm4 | -0.196 | 6.62E-02 | 1.15 |
| ENSRNOG00000048981 | Ahsa1 | -0.186 | 9.74E-02 | 1.14 |
| ENSRNOG00000010473 | Cand2 | -0.186 | 9.77E-02 | 1.14 |
| ENSRNOG00000057284 | Cenpb | -0.186 | 7.65E-02 | 1.14 |
| ENSRNOG00000001517 | Pdk1 | -0.166 | 9.48E-02 | 1.12 |
| ENSRNOG00000009466 | Unc45b | -0.166 | 4.91E-02 | 1.12 |
| ENSRNOG00000020266 | Eef2 | -0.147 | 4.05E-02 | 1.11 |
| ENSRNOG00000014525 | Hspd1 | -0.147 | 7.57E-02 | 1.11 |
| ENSRNOG00000018090 | Ppp6r1 | -0.121 | 9.32E-02 | 1.09 |
|  |  |  |  |  |
| **Female Up Regulated** | | | | |
|  | Gene Symbol | FoldChange (Log_2_) | padj | effect |
| ENSRNOG00000017952 | NA | 0.147 | 4.50E-02 | 1.11 |
| ENSRNOG00000010286 | Cast | 0.194 | 8.30E-02 | 1.14 |
| ENSRNOG00000030118 | Msn | 0.212 | 2.39E-02 | 1.16 |
| ENSRNOG00000059016 | Tspan12 | 0.217 | 8.30E-02 | 1.16 |
| ENSRNOG00000024250 | Wwc3 | 0.222 | 2.90E-02 | 1.17 |
| ENSRNOG00000015380 | Jup | 0.225 | 4.05E-02 | 1.17 |
| ENSRNOG00000010370 | Tnip1 | 0.227 | 3.96E-02 | 1.17 |
| ENSRNOG00000017752 | Mccc2 | 0.229 | 1.77E-02 | 1.17 |
| ENSRNOG00000008236 | Decr1 | 0.236 | 4.38E-02 | 1.18 |
| ENSRNOG00000046949 | Kcnb1 | 0.266 | 4.83E-02 | 1.20 |
| ENSRNOG00000060979 | Hspa13 | 0.273 | 5.95E-02 | 1.21 |
| ENSRNOG00000020173 | Tie1 | 0.280 | 8.01E-02 | 1.21 |
| ENSRNOG00000024402 | Focad | 0.282 | 4.36E-02 | 1.22 |
| ENSRNOG00000040257 | Chmp2b | 0.285 | 1.73E-02 | 1.22 |
| ENSRNOG00000054257 | Adam10 | 0.287 | 6.62E-02 | 1.22 |
| ENSRNOG00000051706 | Tep1 | 0.290 | 1.63E-02 | 1.22 |
| ENSRNOG00000002255 | Fam162a | 0.291 | 4.64E-03 | 1.22 |
| ENSRNOG00000005067 | Zfp36l2 | 0.291 | 4.90E-02 | 1.22 |
| ENSRNOG00000018276 | Mgat4a | 0.296 | 6.21E-02 | 1.23 |
| ENSRNOG00000012563 | Arhgap29 | 0.296 | 8.00E-02 | 1.23 |
| ENSRNOG00000013135 | Ptpn12 | 0.299 | 2.51E-02 | 1.23 |
| ENSRNOG00000061893 | Leng1 | 0.299 | 8.70E-02 | 1.23 |
| ENSRNOG00000023352 | Fam78a | 0.300 | 8.30E-02 | 1.23 |
| ENSRNOG00000004132 | Lasp1 | 0.301 | 4.88E-02 | 1.23 |
| ENSRNOG00000046996 | Pea15 | 0.305 | 2.51E-02 | 1.24 |
| ENSRNOG00000032463 | Rap1a | 0.307 | 3.15E-02 | 1.24 |
| ENSRNOG00000011245 | Arhgap18 | 0.311 | 2.28E-02 | 1.24 |
| ENSRNOG00000001706 | Kalrn | 0.324 | 5.29E-02 | 1.25 |
| ENSRNOG00000060479 | Sp3 | 0.332 | 2.55E-02 | 1.26 |
| ENSRNOG00000060349 | Fam3c | 0.336 | 5.70E-02 | 1.26 |
| ENSRNOG00000060381 | Col15a1 | 0.336 | 7.42E-02 | 1.26 |
| ENSRNOG00000007600 | Igsf1 | 0.338 | 9.07E-02 | 1.26 |
| ENSRNOG00000010744 | Nrp1 | 0.349 | 2.35E-02 | 1.27 |
| ENSRNOG00000004110 | Trib2 | 0.352 | 3.31E-02 | 1.28 |
| ENSRNOG00000016219 | Vnn1 | 0.359 | 8.00E-02 | 1.28 |
| ENSRNOG00000013324 | Cdh5 | 0.359 | 1.15E-02 | 1.28 |
| ENSRNOG00000052296 | Shank3 | 0.362 | 4.36E-02 | 1.29 |
| ENSRNOG00000018483 | Smad1 | 0.364 | 3.26E-02 | 1.29 |
| ENSRNOG00000021380 | Fads6 | 0.365 | 1.08E-02 | 1.29 |
| ENSRNOG00000002045 | Anxa3 | 0.371 | 1.27E-02 | 1.29 |
| ENSRNOG00000056836 | Cav1 | 0.375 | 5.61E-05 | 1.30 |
| ENSRNOG00000019265 | Pcdh12 | 0.375 | 7.42E-02 | 1.30 |
| ENSRNOG00000057903 | NA | 0.376 | 8.49E-02 | 1.30 |
| ENSRNOG00000008369 | Gimap4 | 0.376 | 6.90E-02 | 1.30 |
| ENSRNOG00000042978 | Ncald | 0.387 | 6.41E-02 | 1.31 |
| ENSRNOG00000021179 | Naa40 | 0.392 | 1.52E-02 | 1.31 |
| ENSRNOG00000002653 | Kcnk2 | 0.393 | 2.51E-02 | 1.31 |
| ENSRNOG00000056524 | Abcd1 | 0.401 | 2.39E-03 | 1.32 |
| ENSRNOG00000020546 | Lipe | 0.411 | 9.74E-02 | 1.33 |
| ENSRNOG00000003747 | Asna1 | 0.420 | 8.28E-02 | 1.34 |
| ENSRNOG00000018462 | Rabep2 | 0.428 | 2.46E-02 | 1.35 |
| ENSRNOG00000034116 | Gk | 0.429 | 1.36E-02 | 1.35 |
| ENSRNOG00000017093 | Pxdc1 | 0.436 | 2.52E-02 | 1.35 |
| ENSRNOG00000004968 | Ncapg2 | 0.437 | 2.90E-02 | 1.35 |
| ENSRNOG00000018858 | Myct1 | 0.443 | 3.57E-02 | 1.36 |
| ENSRNOG00000009113 | Marcksl1 | 0.446 | 8.88E-02 | 1.36 |
| ENSRNOG00000022697 | Clec14a | 0.447 | 2.91E-04 | 1.36 |
| ENSRNOG00000013656 | Lpar1 | 0.448 | 6.91E-02 | 1.36 |
| ENSRNOG00000059956 | Bcl6b | 0.449 | 9.74E-02 | 1.37 |
| ENSRNOG00000005386 | Kitlg | 0.454 | 4.84E-03 | 1.37 |
| ENSRNOG00000008904 | Fli1 | 0.456 | 4.50E-02 | 1.37 |
| ENSRNOG00000012929 | Wsb1 | 0.457 | 2.52E-02 | 1.37 |
| ENSRNOG00000057713 | Cav2 | 0.467 | 2.98E-04 | 1.38 |
| ENSRNOG00000004147 | Abca8a | 0.471 | 5.13E-02 | 1.39 |
| ENSRNOG00000030763 | Dpp4 | 0.474 | 5.13E-02 | 1.39 |
| ENSRNOG00000022946 | Slc22a3 | 0.483 | 6.32E-02 | 1.40 |
| ENSRNOG00000018681 | Nes | 0.487 | 7.40E-02 | 1.40 |
| ENSRNOG00000012302 | Gucy1a3 | 0.487 | 3.55E-02 | 1.40 |
| ENSRNOG00000002141 | Cd200 | 0.492 | 5.89E-02 | 1.41 |
| ENSRNOG00000046005 | Scd2 | 0.498 | 7.83E-03 | 1.41 |
| ENSRNOG00000031232 | Nrp2 | 0.508 | 1.89E-02 | 1.42 |
| ENSRNOG00000036604 | Ifit2 | 0.519 | 1.86E-02 | 1.43 |
| ENSRNOG00000020467 | Nrep | 0.532 | 7.42E-02 | 1.45 |
| ENSRNOG00000059750 | NA | 0.548 | 9.45E-02 | 1.46 |
| ENSRNOG00000049743 | Gstm5 | 0.548 | 7.99E-02 | 1.46 |
| ENSRNOG00000017496 | Cnp | 0.551 | 4.84E-03 | 1.47 |
| ENSRNOG00000022615 | Tp53i13 | 0.557 | 6.87E-02 | 1.47 |
| ENSRNOG00000032596 | RT1-T24-1 | 0.582 | 2.43E-02 | 1.50 |
| ENSRNOG00000013793 | C1qtnf9 | 0.584 | 5.27E-04 | 1.50 |
| ENSRNOG00000054614 | NA | 0.604 | 3.97E-02 | 1.52 |
| ENSRNOG00000058357 | Gstm5 | 0.605 | 4.05E-02 | 1.52 |
| ENSRNOG00000008167 | Abhd6 | 0.620 | 5.29E-03 | 1.54 |
| ENSRNOG00000010584 | Tmem123 | 0.623 | 1.45E-05 | 1.54 |
| ENSRNOG00000051056 | NA | 0.623 | 1.60E-02 | 1.54 |
| ENSRNOG00000006670 | Rai2 | 0.625 | 7.81E-03 | 1.54 |
| ENSRNOG00000012183 | Glrx | 0.681 | 1.52E-02 | 1.60 |
| ENSRNOG00000060896 | NA | 0.684 | 7.24E-02 | 1.61 |
| ENSRNOG00000060518 | NA | 0.691 | 3.15E-02 | 1.61 |
| ENSRNOG00000055956 | NA | 0.692 | 7.40E-02 | 1.62 |
| ENSRNOG00000054945 | NA | 0.710 | 1.60E-02 | 1.64 |
| ENSRNOG00000062252 | NA | 0.738 | 9.93E-05 | 1.67 |
| ENSRNOG00000011668 | Nfil3 | 0.785 | 2.45E-02 | 1.72 |
| ENSRNOG00000004307 | Tor3a | 0.864 | 1.36E-02 | 1.82 |

Table S3. Significant genes (FDR<0.01) for Males only.

| **Male Down Regulated** | | | | |
| --- | --- | --- | --- | --- |
|  | Gene Symbol | Fold Change (Log_2_) | padj | effect |
| ENSRNOG00000043451 | Spp1 | -3.358 | 4.38E-03 | 10.25 |
| ENSRNOG00000020837 | Cd300lg | -1.353 | 2.33E-04 | 2.55 |
| ENSRNOG00000062287 | NA | -1.104 | 3.28E-02 | 2.15 |
| ENSRNOG00000020277 | Cntnap1 | -1.070 | 2.93E-02 | 2.10 |
| ENSRNOG00000013973 | Lcn2 | -1.061 | 3.59E-03 | 2.09 |
| ENSRNOG00000034289 | NA | -0.998 | 8.67E-02 | 2.00 |
| ENSRNOG00000029543 | Cish | -0.980 | 5.12E-02 | 1.97 |
| ENSRNOG00000016559 | Tm2d2 | -0.879 | 3.28E-03 | 1.84 |
| ENSRNOG00000059927 | NA | -0.576 | 5.10E-02 | 1.49 |
| ENSRNOG00000032733 | NA | -0.559 | 6.42E-02 | 1.47 |
| ENSRNOG00000007875 | Actr6 | -0.552 | 5.15E-02 | 1.47 |
| ENSRNOG00000061102 | Taf9b | -0.550 | 4.11E-04 | 1.46 |
| ENSRNOG00000006589 | Mif | -0.523 | 1.29E-03 | 1.44 |
| ENSRNOG00000003374 | Ccdc85a | -0.501 | 9.25E-03 | 1.42 |
| ENSRNOG00000056076 | Mif | -0.498 | 1.85E-03 | 1.41 |
| ENSRNOG00000048174 | Uqcrq | -0.483 | 8.83E-06 | 1.40 |
| ENSRNOG00000010107 | NA | -0.404 | 6.66E-04 | 1.32 |
| ENSRNOG00000013954 | Alpl | -0.400 | 4.38E-03 | 1.32 |
| ENSRNOG00000020238 | Plekhh3 | -0.399 | 9.74E-02 | 1.32 |
| ENSRNOG00000020213 | Tubg1 | -0.397 | 2.13E-03 | 1.32 |
| ENSRNOG00000007939 | Naprt | -0.396 | 9.74E-02 | 1.32 |
| ENSRNOG00000008056 | Ankrd9 | -0.386 | 1.56E-02 | 1.31 |
| ENSRNOG00000003063 | Phka1 | -0.378 | 1.83E-02 | 1.30 |
| ENSRNOG00000002610 | Carhsp1 | -0.365 | 3.26E-02 | 1.29 |
| ENSRNOG00000015991 | Npr2 | -0.354 | 1.47E-02 | 1.28 |
| ENSRNOG00000049641 | Rab29 | -0.346 | 2.40E-02 | 1.27 |
| ENSRNOG00000013653 | Pdlim7 | -0.343 | 1.85E-02 | 1.27 |
| ENSRNOG00000010635 | Igfbp4 | -0.342 | 3.50E-02 | 1.27 |
| ENSRNOG00000002828 | Tob1 | -0.335 | 1.96E-02 | 1.26 |
| ENSRNOG00000002827 | Rbfox1 | -0.335 | 2.93E-02 | 1.26 |
| ENSRNOG00000049426 | Mmab | -0.334 | 1.91E-02 | 1.26 |
| ENSRNOG00000019219 | Vamp1 | -0.324 | 9.28E-02 | 1.25 |
| ENSRNOG00000025212 | RGD1565131 | -0.316 | 5.56E-02 | 1.25 |
| ENSRNOG00000020060 | Atf5 | -0.314 | 4.94E-02 | 1.24 |
| ENSRNOG00000006699 | Mlh3 | -0.306 | 1.85E-02 | 1.24 |
| ENSRNOG00000059572 | Tsen34 | -0.295 | 4.52E-02 | 1.23 |
| ENSRNOG00000014064 | Ctsh | -0.292 | 5.10E-02 | 1.22 |
| ENSRNOG00000005610 | Yipf4 | -0.282 | 7.96E-02 | 1.22 |
| ENSRNOG00000017737 | Dgkz | -0.279 | 7.72E-02 | 1.21 |
| ENSRNOG00000006901 | Cd320 | -0.279 | 7.86E-02 | 1.21 |
| ENSRNOG00000005618 | Fmc1 | -0.277 | 7.96E-02 | 1.21 |
| ENSRNOG00000057470 | Pla2g12a | -0.274 | 6.01E-02 | 1.21 |
| ENSRNOG00000024568 | Ndufs7 | -0.260 | 8.81E-03 | 1.20 |
| ENSRNOG00000053640 | Kcng2 | -0.260 | 7.96E-03 | 1.20 |
| ENSRNOG00000020029 | Mcrip2 | -0.259 | 7.96E-02 | 1.20 |
| ENSRNOG00000020544 | Sdr39u1 | -0.259 | 7.43E-02 | 1.20 |
| ENSRNOG00000047768 | Lamb2 | -0.251 | 4.52E-02 | 1.19 |
| ENSRNOG00000001142 | Prkab1 | -0.247 | 3.24E-02 | 1.19 |
| ENSRNOG00000011419 | Aldh6a1 | -0.243 | 4.23E-02 | 1.18 |
| ENSRNOG00000002908 | Slc35a4 | -0.230 | 5.83E-02 | 1.17 |
| ENSRNOG00000018935 | RGD1560015 | -0.195 | 5.83E-02 | 1.14 |
| ENSRNOG00000012014 | Fam160b2 | -0.184 | 9.22E-02 | 1.14 |
| ENSRNOG00000011667 | Fastk | -0.169 | 6.08E-02 | 1.12 |
| ENSRNOG00000056870 | Gdi1 | -0.167 | 3.26E-02 | 1.12 |
|  |  |  |  |  |
| **Male Up Regulated** | | | | |
|  | Gene Symbol | Fold Change (Log_2_) | padj | effect |
| ENSRNOG00000000451 | RT1-Ba | 0.839 | 2.83E-03 | 1.79 |
| ENSRNOG00000008074 | Cyp11a1 | 0.740 | 3.31E-02 | 1.67 |
| ENSRNOG00000037198 | Usp18 | 0.737 | 5.59E-03 | 1.67 |
| ENSRNOG00000054633 | NA | 0.703 | 4.52E-02 | 1.63 |
| ENSRNOG00000026048 | Gbf1 | 0.629 | 5.71E-02 | 1.55 |
| ENSRNOG00000058238 | NA | 0.569 | 7.67E-02 | 1.48 |
| ENSRNOG00000022218 | Ifi44 | 0.554 | 2.90E-02 | 1.47 |
| ENSRNOG00000022871 | LOC691170 | 0.488 | 5.10E-02 | 1.40 |
| ENSRNOG00000029267 | LOC103690821 | 0.465 | 2.33E-02 | 1.38 |
| ENSRNOG00000054080 | Cgnl1 | 0.460 | 2.55E-03 | 1.38 |
| ENSRNOG00000032835 | LOC103690821 | 0.448 | 1.96E-02 | 1.37 |
| ENSRNOG00000038654 | LOC103690821 | 0.447 | 6.01E-02 | 1.36 |
| ENSRNOG00000048073 | LOC103690821 | 0.445 | 5.78E-02 | 1.36 |
| ENSRNOG00000001258 | Snx8 | 0.439 | 2.31E-02 | 1.36 |
| ENSRNOG00000009225 | Copz2 | 0.414 | 5.83E-02 | 1.33 |
| ENSRNOG00000027867 | Rexo4 | 0.384 | 9.77E-02 | 1.31 |
| ENSRNOG00000001312 | Pdgfa | 0.366 | 3.99E-02 | 1.29 |
| ENSRNOG00000007988 | Timm22 | 0.365 | 3.46E-02 | 1.29 |
| ENSRNOG00000008246 | Emilin1 | 0.359 | 2.33E-02 | 1.28 |
| ENSRNOG00000033593 | Osbpl9 | 0.356 | 8.26E-02 | 1.28 |
| ENSRNOG00000003599 | Utp3 | 0.348 | 5.08E-02 | 1.27 |
| ENSRNOG00000002171 | NA | 0.346 | 5.82E-02 | 1.27 |
| ENSRNOG00000007432 | Polr3b | 0.344 | 8.57E-02 | 1.27 |
| ENSRNOG00000012609 | Trdn | 0.318 | 1.61E-02 | 1.25 |
| ENSRNOG00000012269 | Lpcat3 | 0.300 | 4.80E-02 | 1.23 |
| ENSRNOG00000004239 | NA | 0.269 | 1.56E-02 | 1.21 |
| ENSRNOG00000045558 | Cd34 | 0.267 | 3.31E-02 | 1.20 |
| ENSRNOG00000055568 | LOC100910754 | 0.265 | 2.07E-02 | 1.20 |
| ENSRNOG00000008659 | Arhgap21 | 0.263 | 3.93E-02 | 1.20 |
| ENSRNOG00000002373 | Akap1 | 0.249 | 3.96E-02 | 1.19 |
| ENSRNOG00000018333 | Rbm6 | 0.245 | 9.22E-02 | 1.19 |
| ENSRNOG00000032776 | Srp54a | 0.221 | 9.01E-02 | 1.17 |
| ENSRNOG00000027491 | Vldlr | 0.221 | 5.10E-02 | 1.17 |
| ENSRNOG00000013876 | Mipep | 0.221 | 6.42E-02 | 1.17 |
| ENSRNOG00000005796 | Ctnna1 | 0.186 | 1.12E-02 | 1.14 |

Figure S1. Principle Component Analysis scatterplot of RNA sequence data for male and female rats prenatally exposed to methamphetamine. The female rat to the far right (circled) was identified as an outlier and remove from analysis.


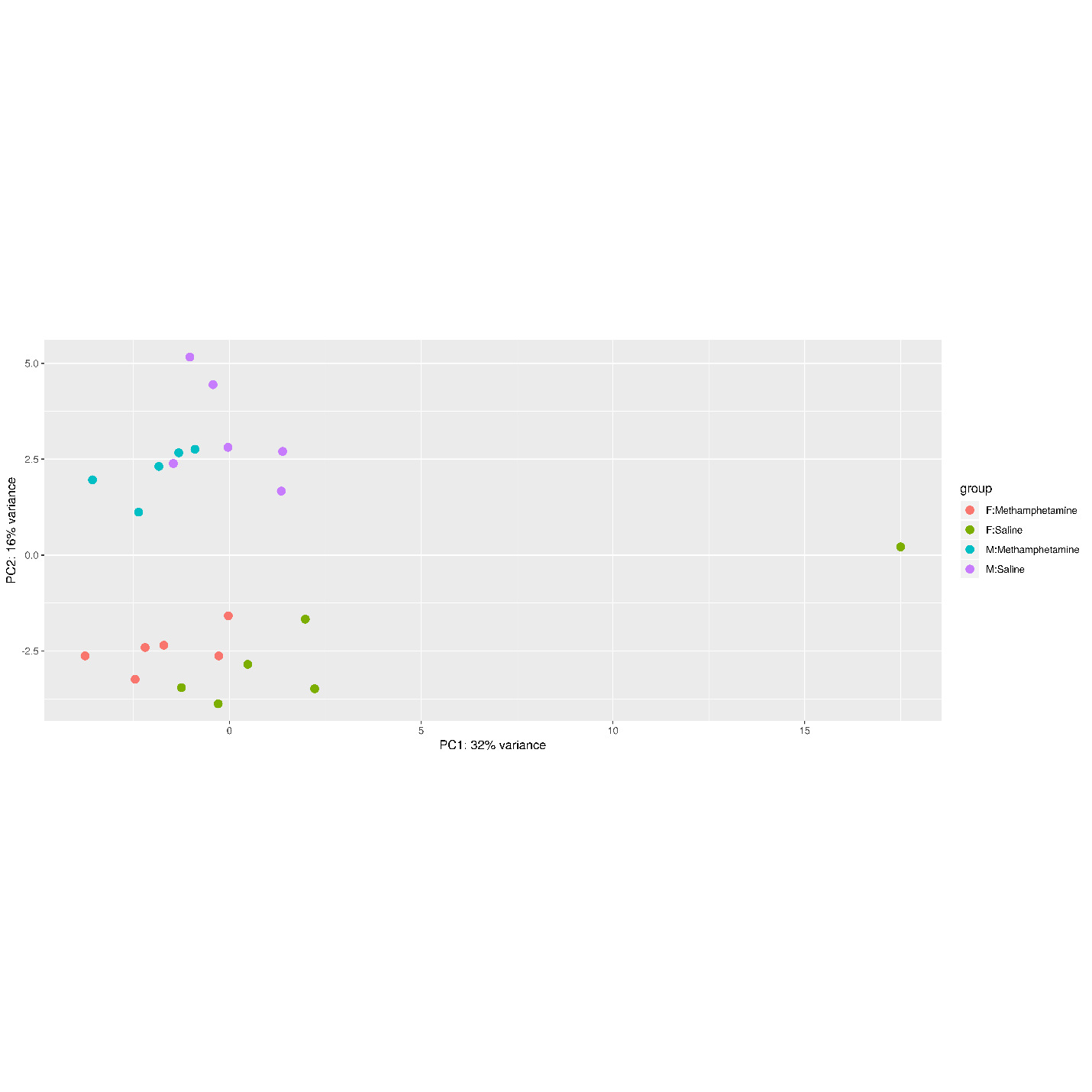


Table S4. Significant genes (FDR<0.01) comparing Saline Males versus Saline Females.

| **Saline Males vs Saline Females** | | | | |  |
| --- | --- | --- | --- | --- | --- |
| **Down Regulated in Males compared to Females** | | | | |  |
|  | Gene Symbol | Fold Change (Log2) | padj | effect |  |
| ENSRNOG00000060793 | Eif2s3 | -0.764 | 6.11E-37 | 1.698 |  |
| ENSRNOG00000051257 | NA | -8.835 | 1.68E-20 | 456.674 | Largely no expression in males |
| ENSRNOG00000057706 | NA | -0.675 | 5.26E-16 | 1.597 |  |
| ENSRNOG00000023383 | Ddx3x | -0.368 | 6.95E-12 | 1.29 |  |
| ENSRNOG00000052721 | Kdm6a | -0.858 | 2.96E-11 | 1.812 |  |
| ENSRNOG00000020266 | Eef2 | -0.256 | 2.65E-06 | 1.194 |  |
| ENSRNOG00000061910 | Igfbp3 | -0.972 | 6.20E-06 | 1.961 |  |
| ENSRNOG00000003330 | Acsf2 | -0.379 | 4.79E-05 | 1.3 |  |
| ENSRNOG00000049385 | Adamtsl4 | -0.472 | 6.56E-05 | 1.387 |  |
| ENSRNOG00000013451 | Drosha | -0.364 | 1.17E-04 | 1.287 |  |
| ENSRNOG00000001276 | Pcnt | -0.473 | 1.88E-04 | 1.388 |  |
| ENSRNOG00000022772 | Prickle1 | -0.39 | 7.50E-04 | 1.311 |  |
| ENSRNOG00000007896 | Klhl38 | -0.489 | 9.00E-04 | 1.404 |  |
| ENSRNOG00000019834 | Hsp90ab1 | -0.315 | 9.00E-04 | 1.244 |  |
| ENSRNOG00000018400 | Golm1 | -0.456 | 1.90E-03 | 1.372 |  |
| ENSRNOG00000046848 | PCOLCE2 | -0.592 | 2.92E-03 | 1.508 |  |
| ENSRNOG00000010103 | Eif4b | -0.239 | 3.50E-03 | 1.181 |  |
| ENSRNOG00000016343 | Dkk3 | -0.34 | 6.12E-03 | 1.265 |  |
| ENSRNOG00000020729 | Stc2 | -0.544 | 6.91E-03 | 1.458 |  |
| **Up Regulated in Males compared to Females** | | | | |  |
|  | Gene Symbol | Fold Change (Log2) | padj | effect |  |
| ENSRNOG00000057231 | Ddx3 | 11.097 | 5.18E-30 | 2190.137 | Largely no expression in females |
| ENSRNOG00000060048 | Eif2s3y | 10.549 | 2.81E-27 | 1497.855 | Largely no expression in females |
| ENSRNOG00000062252 | NA | 0.921 | 1.23E-07 | 1.894 |  |
| ENSRNOG00000054945 | NA | 1.167 | 5.05E-07 | 2.246 |  |
| ENSRNOG00000060518 | NA | 1.178 | 1.85E-06 | 2.262 |  |
| ENSRNOG00000060896 | NA | 1.253 | 5.90E-06 | 2.383 |  |
| ENSRNOG00000055956 | NA | 1.259 | 6.93E-06 | 2.393 |  |
| ENSRNOG00000004517 | Igf1 | 0.803 | 2.19E-04 | 1.745 |  |
| ENSRNOG00000057713 | Cav2 | 0.473 | 2.70E-04 | 1.388 |  |
| ENSRNOG00000008831 | Hcn2 | 0.283 | 1.11E-03 | 1.217 |  |
| ENSRNOG00000033615 | ND3 | 0.555 | 1.36E-03 | 1.47 |  |
| ENSRNOG00000054614 | NA | 0.786 | 1.98E-03 | 1.724 |  |
| ENSRNOG00000009310 | Nmd3 | 0.3 | 3.36E-03 | 1.231 |  |
| ENSRNOG00000029886 | Hba1 | 3.541 | 3.50E-03 | 11.644 |  |
| ENSRNOG00000009113 | Marcksl1 | 0.632 | 4.04E-03 | 1.549 |  |
| ENSRNOG00000030700 | COX3 | 0.413 | 5.19E-03 | 1.332 |  |
| ENSRNOG00000056836 | Cav1 | 0.302 | 5.56E-03 | 1.233 |  |
| ENSRNOG00000013102 | Entpd2 | 0.531 | 5.74E-03 | 1.445 |  |
| ENSRNOG00000017952 | NA | 0.182 | 6.91E-03 | 1.134 |  |
| ENSRNOG00000022697 | Clec14a | 0.374 | 8.17E-03 | 1.296 |  |
| ENSRNOG00000061299 | LOC100134871 | 3.534 | 9.05E-03 | 11.582 |  |
| ENSRNOG00000015519 | Ces1d | 0.793 | 9.52E-03 | 1.733 |  |
| ENSRNOG00000058105 | Hbb | 3.468 | 9.52E-03 | 11.067 |  |
